# Supplementary material for: Replicating Dynamic Immune Responses at Single‐Cell Resolution within a Microfluidic Human Skin Equivalent
Source: Adv Sci (Weinh). 2025 Jan 21;12(10):2415717. doi: 10.1002/advs.202415717 (PMC11905070; doi:10.1002/advs.202415717)
Supplement: Supplementary file 1 — Supporting Information [file ADVS-12-2415717-s003.pdf]

ADVANCED SCIENCE
Open Access

Supporting Information

for *Adv. Sci.*, DOI 10.1002/advs.202415717

Replicating Dynamic Immune Responses at Single-Cell Resolution within a Microfluidic Human Skin Equivalent

*Sarah A. Hindle, Holly Bachas Brook, Alexandra Chrysanthou, Emma S. Chambers, Matthew P. Caley and John T. Connelly**

Supporting Information

Replicating dynamic immune responses at single-cell resolution within a microfluidic human skin equivalent

Sarah A. Hindle, Holly Bachas Brook, Alexandra Chrysanthou, Emma S. Chambers, Matthew P. Caley, John T. Connelly[*]

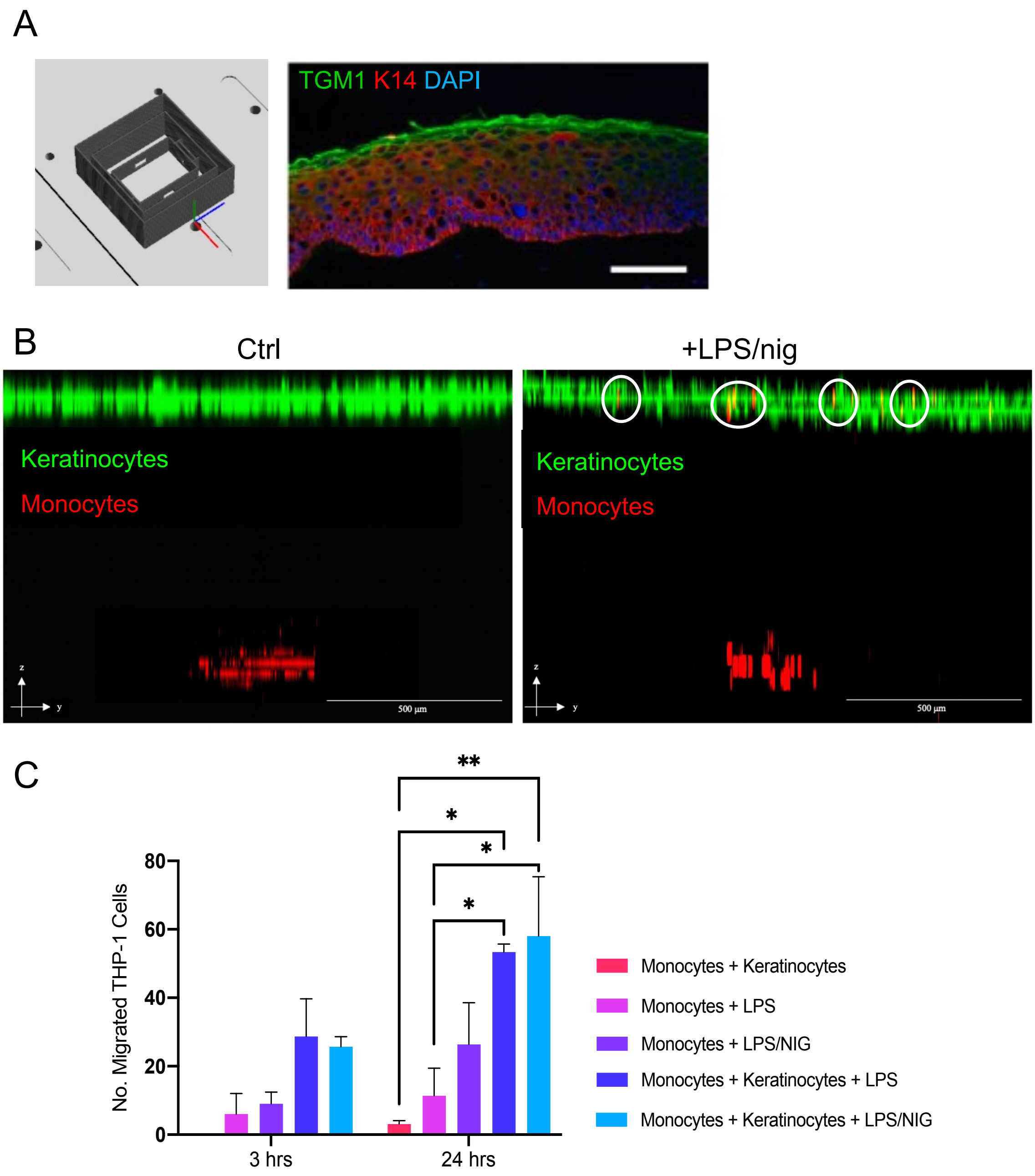

Figure S1: Development of microfluidic HSE and analysis of monocyte migration. (A) 3D CAD design of chamber for air-liquid interface cultures and immunofluorescence image of K14 and tranglutaminase-1 in stratified epidermal layers of HSEs at day 14. (B) Cross-sectional views of confocal Z-stacks for HSEs containing fluorescently labelled keratinocytes and THP-1 monocytes, 24 h after control or LPS/nig treatment. (C) Quantification of monocytes per field of view in the epidermis in HSEs with monocytes alone or monocytes and keratinocytes at 3h and 24 h after control, LPS, or LPS/nig treatment. Data represent mean ± SEM of N=3 experiments, *p<0.05, **p<0.1, ANOVA.

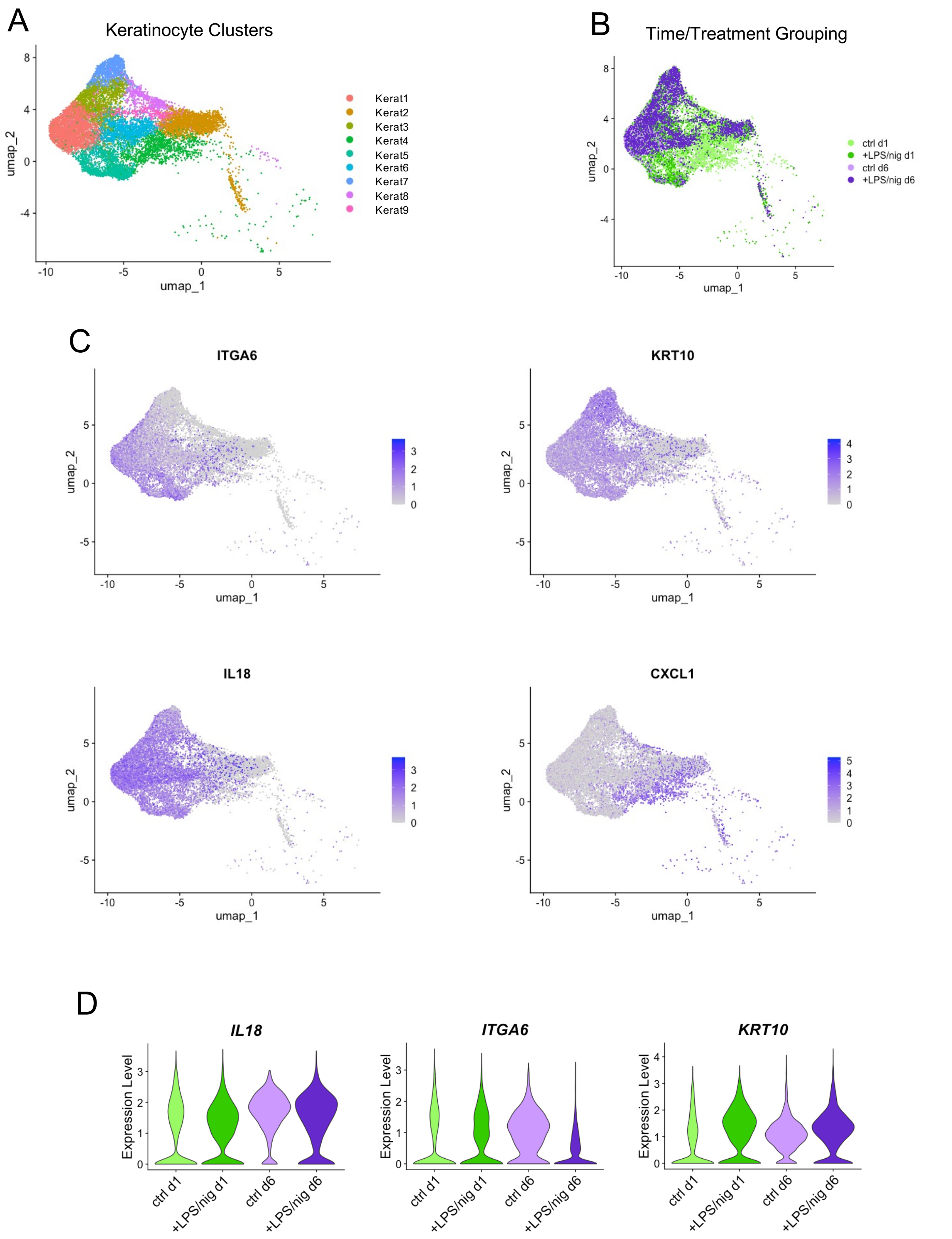

Figure S2: Analysis of keratinocyte clusters. (A) UMAP visualisation of all keratinocyte clusters in the single-cell RNA-seq data set. (B) Feature plot of time point and treatment conditions in keratinocyte clusters. (C) Feature plots for key discriminatory markers for keratinocytes clusters, including differentiation (*ITGA6, KRT10*) and inflammatory genes (*IL18, CXCL1*). (D) Violin plots of *IL18, ITGA6,* and *KRT10* across the experimental conditions.

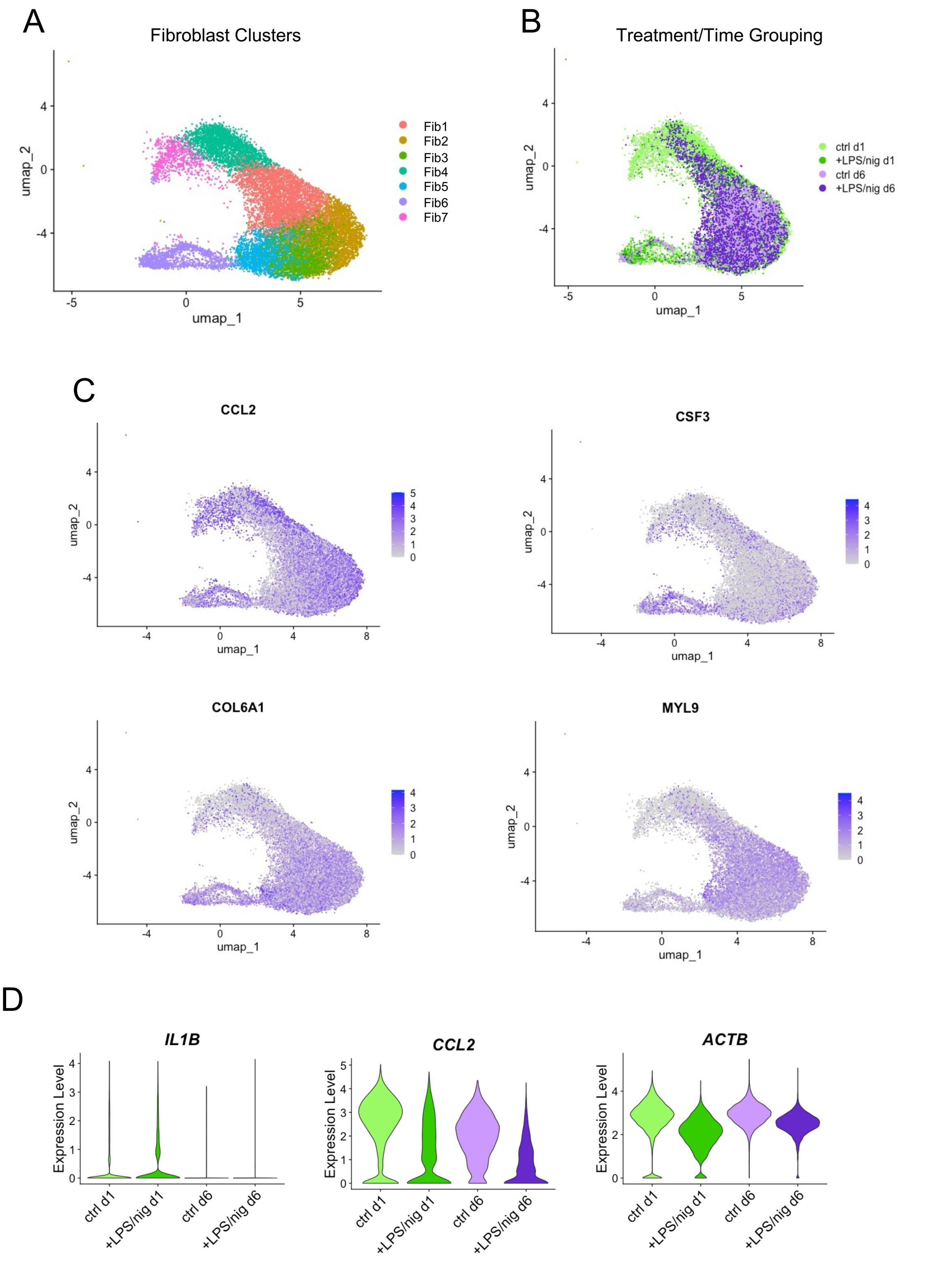

Figure S3: Analysis of fibroblast clusters. (A) UMAP visualisation of all fibroblast clusters in the single-cell RNA-seq data set. (B) Feature plot of time point and treatment conditions in fibroblast clusters. (C) Feature plots for key discriminatory markers for fibroblast clusters, including *CCL2, CSF3, COL6A1, MYL9*. (D) Violin plots of *IL1B, CCL2,* and *ACTB* across the experimental conditions.

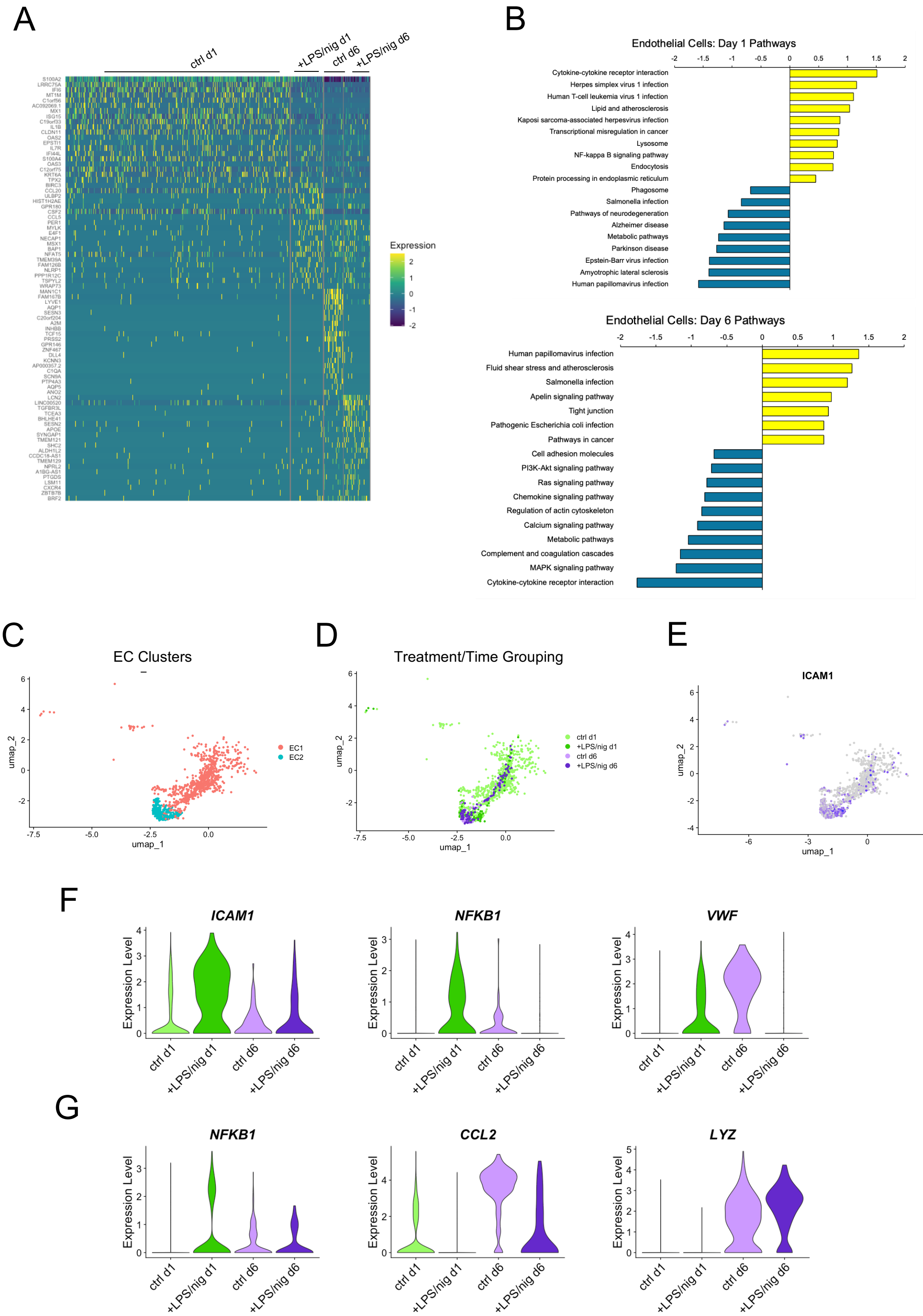

Figure S4: Analysis of EC clusters and pathways and additional monocyte genes. (A) Heatmap of the top 20 differentially expressed genes (DEGs; Log2FC > 1; Padj < 0.05) between experimental conditions for all EC clusters. (B) Gene set enrichment analysis (GSEA; Webgestalt) of EC pathways up and down regulated by LPS/nig at days 1 and 6. Data represent pathways with the top 10 highest and lowest relative enrichment scores. (C) UMAP visualisation of the two EC clusters in the single-cell RNA-seq data set. (D) Feature plot of time point and treatment conditions in EC clusters. (E) Feature plots for *ICAM1*. (F) Violin plots of *ICAM1, NFKB1, and VWF* across the experimental conditions. (G) Violin plots of *NFKB1*, *CCL2*, and *LYZ* in monocytes.

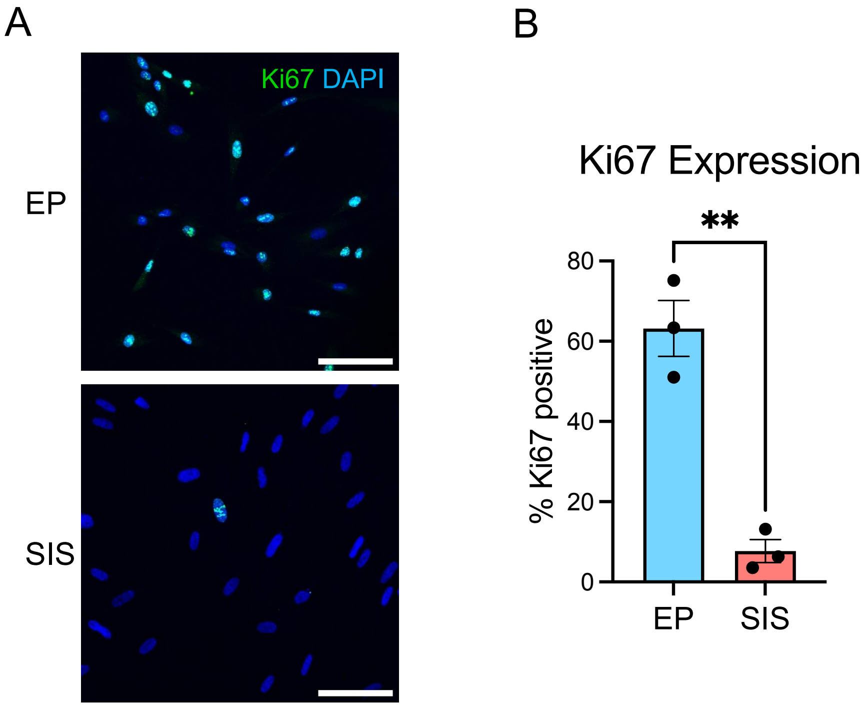

Figure S5: Validation of cell cycle arrest in senescent fibroblasts. (A) Immunofluorescence images of Ki67 in EP and SIS fibroblasts cultured on glass coverslips for 48 h after completion of the H_2O_2 treatment protocol. Scale bar = 100 µm. (B) Quantification of the percentage of Ki67 positive cells in EP and SIS fibroblasts on coverslips. Data represent the mean ± SEM of N=3 experiments, $^{**}p<0.005$, t-test.
